# Targeted genomic analysis of a predominant uncultured marine pelagiphage-host model via microfluidics and semipermeable capsule technology

**DOI:** 10.1093/ismeco/ycaf123

**Published:** 2025-07-17

**Authors:** Manuel Martinez-Garcia, Monica Lluesma-Gomez, Laura Perez-Martin, Esther Rubio-Portillo, Ana Belen Martin-Cuadrado, Francisco Nadal-Molero, Aitana Escolano-Vico, Fernando Santos Sanchez, Victoria Orphan, Josefa Antón

**Affiliations:** Instituto Multidisciplinar para el Estudio del Medio Ramon Margalef, Parque Científico, Edificio Nuevos Institutos, University of Alicante, Ap- Correos 99, E-03690, San Vicente del Raspeig, Alicante, Spain; Department of Physiology, Genetics, and Microbiology, University of Alicante, Carretera de San Vicente s/n, 03080 San Vicente del Raspeig, Alicante, Spain; Instituto Multidisciplinar para el Estudio del Medio Ramon Margalef, Parque Científico, Edificio Nuevos Institutos, University of Alicante, Ap- Correos 99, E-03690, San Vicente del Raspeig, Alicante, Spain; Department of Physiology, Genetics, and Microbiology, University of Alicante, Carretera de San Vicente s/n, 03080 San Vicente del Raspeig, Alicante, Spain; Department of Physiology, Genetics, and Microbiology, University of Alicante, Carretera de San Vicente s/n, 03080 San Vicente del Raspeig, Alicante, Spain; Department of Physiology, Genetics, and Microbiology, University of Alicante, Carretera de San Vicente s/n, 03080 San Vicente del Raspeig, Alicante, Spain; Department of Physiology, Genetics, and Microbiology, University of Alicante, Carretera de San Vicente s/n, 03080 San Vicente del Raspeig, Alicante, Spain; Department of Physiology, Genetics, and Microbiology, University of Alicante, Carretera de San Vicente s/n, 03080 San Vicente del Raspeig, Alicante, Spain; Department of Physiology, Genetics, and Microbiology, University of Alicante, Carretera de San Vicente s/n, 03080 San Vicente del Raspeig, Alicante, Spain; Department of Physiology, Genetics, and Microbiology, University of Alicante, Carretera de San Vicente s/n, 03080 San Vicente del Raspeig, Alicante, Spain; Division of Biology and Biological Engineering, California Institute of Technology, Pasadena, CA 91125, United States; Division of Geological and Planetary Sciences, California Institute of Technology, Pasadena, CA 91125, United States; Instituto Multidisciplinar para el Estudio del Medio Ramon Margalef, Parque Científico, Edificio Nuevos Institutos, University of Alicante, Ap- Correos 99, E-03690, San Vicente del Raspeig, Alicante, Spain; Department of Physiology, Genetics, and Microbiology, University of Alicante, Carretera de San Vicente s/n, 03080 San Vicente del Raspeig, Alicante, Spain

**Keywords:** microfluidics, semipermeable capsules, virus, bacteria, host, genome, Pelagibacter, SAR11, vSAG 37-F6, pelagiphage

## Abstract

Microbes and their viruses drive central biogeochemical cycles on a global scale. Understanding the biology and ecology of virus–host interactions and their impact on ecosystems depends on our ability to develop tools that enable high-throughput screening of ecologically relevant, uncultured virus–host pairs. Viruses infecting Pelagibacterales, the predominant bacteria in surface oceans, have been studied through computational analyses and cultivation efforts. Here, we employ an accessible microfluidics and semi-permeable capsule (SPC) technology to investigate the uncultured pelagiphage vSAG 37-F6–host interactions since it is one of the most abundant and ubiquitous viruses in the marine virosphere. First, we validated this technology using cultured virus–host pairs. Then, marine single cells were microfluidically encapsulated in SPCs, lysed, whole-genome amplified, and screened using fluorescent polymerase chain reaction (PCR) for the presence of a hallmark gene of vSAG 37-F6. Data indicate that ~30% of the targeted cell population (cell fraction ≤0.45 μm) contained the virus vSAG 37-F6-like. A total of ~500 putatively infected cells were sorted, combined, and sequenced. Data showed that most reads (~60%) and assembled genome fragments (~85%) were identified as viral, indicating that the sorted host cells were likely in the final stages of infection. Two major viral clusters were detected: one corresponding to vSAG 37-F6 and another mixed viral cluster consisting of cyanophages, pelagiphages, and vibriophages. A significant proportion of total reads (~20%) were assigned to *Pelagibacter* spp. TMED287, a bacterium reported to be abundant in the Mediterranean Sea. This flexible microfluidic-SPC technology holds enormous potential for exploring uncultured microbial and viral communities across various perspectives and microbiology fields.

## Introduction

Microbes and their viruses are the most abundant and diverse life forms in the biosphere and drive central biogeochemical cycles on a global scale [1, 2]. Despite advances in culturomics [3–5], many ecologically relevant microbial virus–host pairs are yet to be cultured. Metagenomics and single-cell genomics (SCGs) have been pivotal in characterizing the uncultured majority of microbes and viruses, significantly expanding our ability to explore complex microbial ecosystems and revealing their functional diversity and taxonomic composition [6–13].

Understanding the biology and ecology of these virus–host interactions and their influence on the ecosystem also depends on our ability to develop experimental techniques that enable high-throughput screening of relevant uncultured virus–host pairs, complementing metagenomic data. Viruses play an essential role in controlling bacterial populations and those infecting Pelagibacterales (SAR11), the dominant bacteria in surface oceans, have been studied by metagenomics and by cultivation techniques [14–16]. Advances in empirical methodologies and bioinformatic approaches have been paramount in linking uncultured viruses to putative hosts by identifying genomic signatures indicative of virus–host associations [6, 17–19].

**Figure 1:**
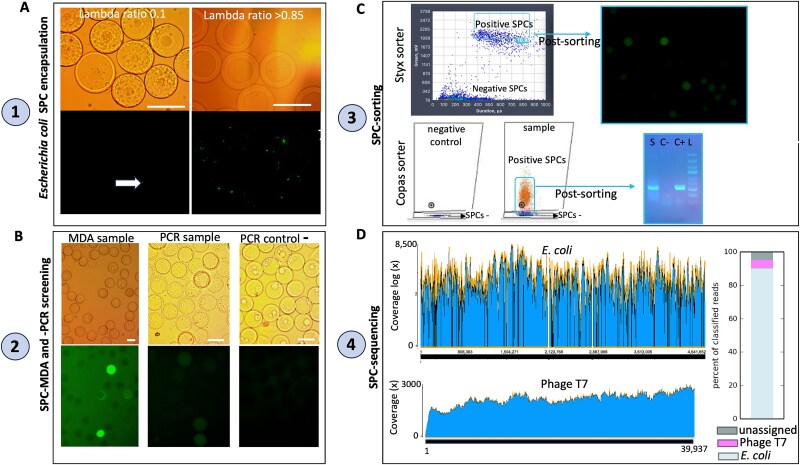
Validation of microfluidic SPC technology with *Escherichia coli* infected with bacteriophage T7. Numbers from 1 to 4 refers to the workflow order of the technique. (A) SPC encapsulation of an infected culture of *E. coli* with phage T7 at the initial infection stage using two different lambda rates: 0.1 used in downstream experiments (90% of SPCs theoretically empty; arrow points to a single cell stained with SYTO9 encapsulated in an SPC) and for comparison, a lambda ratio >0.85 with most of SPC occupied with multiple cells stained with SYTO9 (upper images from bright field microscopy and bottom images from epifluorescence microscopy). Scale bar is 80 μm. (B) Left image: MDA results from an infected culture of *E. coli* with phage T7 encapsulated at a lambda rate of 0.1. A fraction of post MDA-SPCs (top image; bright field microscopy) are labelled with SYTO 9 for visualization under an epifluorescence microscopy (bottom image). Center image: the rest of MDA-SPC sample underwent fluorescence PCR screening with specific primers labeled at 5′ with fluorochrome AlexaFluo488 targeting a specific gene of bacteriophage T7 genome. Right image: a negative control of fluorescence PCR screening of SPCs (i) encapsulating sterile ultrapure water and (ii) screening the sample with a fluorescently labeled primer set for a different virus (i.e. specific primers for marine virus vSAG 37-F6). Scale bar is 70 μm. (C) Detection and sorting of positive PCR-SPCs with microfluidic sorter Styx (Atrandi) and post-visualization of sorted SPCs (upper image) and Copas sorter. For the latter, corroboration of specific size of PCR amplicons from the targeted viral gene of T7 was checked in a conventional agarose electrophoresis gel (S: sample, C−: negative PCR control, C+: positive PCR control using bacteriophage T7 DNA, L: ladder 1 kb). (D) Mapping of sequencing reads with bowtie program against *E. coli* host (upper image) genome and bacteriophage T7 (bottom image). A bar plot showing taxonomic assignment of raw unassembled data is shown (right image).

## Results and discussion

Here, we employed a commercial, accessible microfluidics, and semi-permeable capsules (SPCs) technology [20–22] to study virus–host interactions in one of the most abundant and ubiquitous virus models in the marine viriosphere: the uncultured virus vSAG 37-F6, discovered through single-virus genomics and known to infect uncultured *Pelagibacter* spp. [23]. The microfluidic SPC technology used here allows for high-throughput screening of tens or hundreds or thousands of individual cells in SPCs without loss of single-cell compartmentalization [20], using a flexible, modular workflow that supports multi-step molecular analysis. In brief, single cells were encapsulated in SPCs to achieve a lambda ratio of 0.1 (10.39% of SPCs occupied with a single cell; with a frequency of an SPC containing multiple single cells of **~**0.5%). Cell were then lysed and whole-genome amplified by multiple-displacement amplification (MDA) following SCGs standards [10, 24]. After this process, each SPC contained amplified genomic DNA derived from a single cell. PCR screening of SPCs was then performed using specific 5′-fluorescently labeled primers targeting a viral hallmark gene indicative of viral presence in SPCs. DNA fragments ~>500 bp, such as MDA or PCR products obtained in our study, are retained inside SPCs. Finally, positive fluorescently labeled SPCs, indicative of a cell infected with the target virus, were sorted, subjected to a second round of MDA to generate enough DNA, and bulk DNA from sorted SPC was sequenced, and analyzed.

We first successfully validated SPC microfluidic technology for virus–host interaction using a known cultured virus–host model (*Escherichia coli* K12 infected with bacteriophage T7; Fig. 1). See a complete discussion of validation of SPC in supplementary material (Supplementary Fig. S1 and Supplementary Table S1). Data indicate that the genome recovery was complete for the virus and nearly complete for the host (>99% breath coverage and nucleotide identity; Fig. 1).

**Figure 2:**
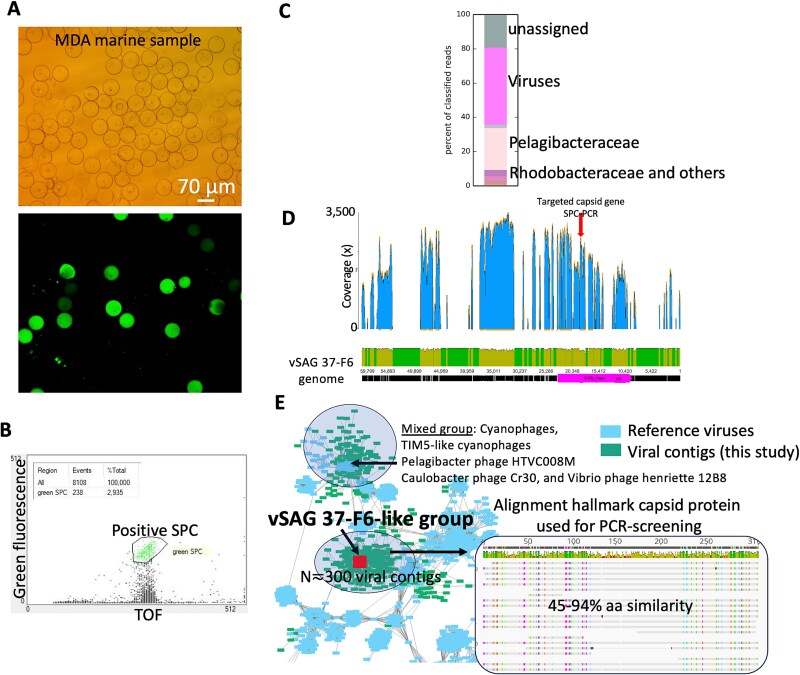
Analysis of virus–host interaction throughout microfluidic SPC technology for the uncultured virus model pelagiphage vSAG 37-F6. (A) Multiple-displacement amplification results of a surface seawater sample encapsulated at a lambda ratio of 0.1. Lysis and MDA were performed following single cell genomic protocols (see Methods in Supplementary Material for details). Top image shows SPCs that underwent MDA amplification. MDA product within SPC is stained with SYTO9 and visualized with epifluorescence microscopy (bottom image). (B) Detection of positive PCR-SPCs screened with specific primers targeting a hallmark capsid gene (ORF 9) of vSAG 37-F6, previously used in other studies for monitoring abundance and presence of this virus. TOF depicts (relative units) sizes of particle. (C) Once positive SPCs (*n* = 500) were sorted, they underwent a second round of MDA to generate enough DNA for Illumina sequencing. Bar plot shows taxonomic Kaiju assignment of reads obtained from bulk sequencing of DNA recovered from 500 sorted positive SPCs. Within “others” category, we found <3% of reads assigned to *Moraxella* spp., which is a potential contamination from manipulation or microfludic chip design. (D) Chart illustrates mapping results of unassembled reads against the genome of virus vSAG 37-F6 recovered by single virus genomics in 2017 from a sampling site located more than 500 km away from the location studied in this survey. (E) Viral protein sharing network. Nodes indicate viral assembled contigs recovered in this study (green color) and reference marine and non-marine viruses from RefSeq in NCBI Genbank. Edges between nodes indicate a statistically significant weighted pairwise similarity between the protein profiles of each node (see Methods and ref. [27]) with similarity scores ≥1. Viral clusters are determined by applying the Markov cluster algorithm to the edges. vSAG 37-F6 is indicated by a red square. Amino acid alignment of the hallmark capsid protein (ORF 9) used for PCR screening of infected cells is shown in the insert panel.

Following the protocol validated with the culture model, microfluidic SPCs technology was then applied to target uncultured cells infected with the lytic uncultured pelagiphage vSAG 37-F6, which is one of the most abundant, (micro)-diverse, and cosmopolitan marine viruses [23, 25–29]. Despite its ubiquity, the recovery and analysis of vSAG 37-F6 virus–host interactions have remained largely elusive. To target uncultured *Pelagibacter* cells infected with virus vSAG 37-F6, we used as a reporter of viral infection the presence of a hallmark capsid gene of virus vSAG 37-F6, previously identified as the most abundant viral protein in marine ecosystems [27], as detected through viral proteomics [30].

Surface seawater was collected at Cape Huertas (Mediterranean Sea), a site widely studied for the presence and seasonal dynamics of vSAG 37-F6 [29]. Seawater was filtered through 0.45 μm (see Supplementary material for more details). SAR11 cells and other marine species are highly enriched in the fraction of bacterioplankton cells that pass through 0.45-μm filters [31–33]. Small cells (Supplementary Fig. S2) were encapsulated in SPCs to a lambda ratio of 0.1, ensuring a low probability of co-occurrence of two cells in the same SPC (≈0.5%). Then, lysis, MDA, and PCR screening with specific primers for the hallmark capsid gene of virus vSAG 37-F6 and viral relatives were performed (Fig. 2A; Supplementary Fig. S3). Furthermore, a variety of six different controls were implemented to confirm that the observed fluorescence signal from positive SPCs were indeed only detected in the presence of the target virus (see Supplementary material for more details and Supplementary Fig. S4).

Data indicated that ~2.9% of SPCs screened by PCR contained the virus vSAG 37-F6 (see the results from one of the run that resulted in 238 positive SPCs out of 8108 screened SPCs; Fig. 2B). If only ~10% of SPCs contained single cells (~810 SPCs), our results suggest that close to 30% of the targeted cell population (cell fraction ≤0.45 μm) was putatively infected with vSAG 37-F6. Providing accurate*,* absolute abundances of uncultured virus–host pairs in nature is ecologically relevant for ecosystem modeling, yet remains a methodological challenge [34, 35]. In the sub-Antarctic Pacific Ocean, results from the polony method indicates high cyanophage infection levels up to 26.2%, similar to our data.

After PCR screening, using the same aliquot sample for estimating abundance of vSAG 37-F6 in cell population, a total of ~500 positive fluorescently labeled SPCs were sorted with the Copas instrument (Fig. 2B; see controls and blanks in Supplementary Fig. S4), SPCs were dissolve,d, and the obtained bulk DNA was sequenced. Analysis of unassembled sequencing data from sorted SPCs (Supplementary Table S1) showed that ~60% of the sequenced reads were assigned to viruses (Fig. 2C). Specific mapping of sequenced reads to the vSAG 37-F6 genome showed that multiple hallmark genes commonly present in vSAG 37-F6 genome including its capsid gene, were present in sorted SPCs (Fig. 2D). The full mapping of vSAG 37-F6 genome was not obtained (Fig. 2D). This is not surprising given the previously described genetic and genomic (micro)-diversity and co-occurrence of different species and strains of this virus in the same sample [28, 29]. The co-occurrence of up to ~1500 distinct viral strains (with >95% nucleotide identity) and around 30 related species (with 80%–95% nucleotide identity) has been reported in a single seawater sample [28]. Indeed, a viromic fragment recruitment analysis performed in a recent study [29] in the same sampling site and season addressed here, demonstrated that several genomic regions of vSAG 37-F6 genome were not present. Furthermore, when searching for the prokaryotic host DNA signals in SPCs, a large proportion of total sequenced reads (~30%) belonged to Alphaproteobacteria, particularly *Pelagibacter* spp. TMED287 (~20%), a metagenome-assembled genome reported to be abundant in the Mediterranean Sea [36] (Fig. 2D). Putative contamination in the unassembled dataset (e.g. *Moraxella* likely from human source), was minimal (<3%; Fig. 2C). Therefore, our data suggest that at the time of sampling, a different array of vSAG 37-F6-related species—distinct from that originally described in 2017 in Blanes Bay Microbial Observatory—were putatively infecting the sorted cell population of SAR11.

A detailed analysis of the assembled dataset from the SPCs (*n* = 2109 contigs ≥500 bp; 6.3 Mbp, average frequency of guanine-cytosine (GC) of 39%) revealed that a significant proportion of contigs were viral contigs belonging to the *Cardioviral* class (*n* = 1321; 5.36 Mbp; average GC 38%; longest contig 84 kb; 105 contigs ≥10 kb; Fig. 2E). Two large, highly connected viral subnetworks were found (Fig. 2E). One viral cluster containing the highest number of nodes or viral contigs (*n* = ~345) was related with vSAG 37-F6 genome, which was placed at the center of this cluster highly connected with the rest of nodes. We identified 20 viral contigs within this subnetwork that carried the definitive hallmark capsid gene (45%–94.4% of amino acid similarity; Fig. 2E; insert panel) in addition to other orthologous genes of vSAG 37-F6 (average query coverage 76%, average amino acid identity 53.5%). Furthermore, in this large subnetwork, no other reference marine viruses except vSAG 37-F6 was found. Considering the largest viral contigs were all related to vSAG 37-F6 (from 10 to 44 kb) in this subnetwork, it was interesting to detected that <50% of the genes were shared with vSAG 37-F6. This suggests that the genomic mosaicism and variability of this virus is high considering the very low cell population size studied (500 cells). The other large viral subnetwork consisted of different types of phages including cyanophage-like [37], Pelagiphage HTVC009M, *Caulobacter* and *Vibrio* phages. Other singleton viral contigs (mostly <1 kb) with no connections to other viruses were also obtained in the viral network; these may represent small genome fragments of larger viral genomes of the network (i.e. a partial fragmented genome assembly).

A low proportion of non-viral sequences (14.9%; 0.94 Mbp of the total 6.3 Mbp assembled data), mostly consisting of small, fragmented contigs (<1 kb), was tentatively assigned to the cellular fraction. Due to the high fragmentation and low completeness (<5%), we were unable to reconstruct any reliable genome bins. Recently, it was demonstrated that pelagiphage-infection of SAR11 cells during a phytoplankton bloom resulted in the formation of zombie cells, which are devoid of any detectable ribosomal RNA [16]. Two hypothesis have been debated: one suggests that such a major cell transformation may be beneficial for preventing the synthesis of phage proteins upon infection making the cells resistant and persistent. Alternatively, phage infection may induce RNA degradation to recycle ribonucleotides for phage genome synthesis [16]. In line with that, a previous study on virus vSAG 37-F6 and its close viral relatives reported a gene putatively involved in recycling of dNTPs (gene initially annotated as “genome maintenance exonuclease 1” and recently renamed as Anobiidae exonuclease) [23]. At least 21 viral contigs obtained in this study were related to vSAG 37-F6 contained this type of exonuclease. Given the minimal detection of host DNA, our data suggest that vSAG 37-F6 phages were active in killing host cells, and that SPC sorting likely occurred when the host cells were in the final stages of infection. However, it is also possible that the very low completeness of host genome may have been due to the MDA genome coverage bias, which is a common problem during WGA [38], which may have preferentially amplified small, circular, or high copy viral genomes. Future improvements to this workflow could include the implementation of primary template-direct amplification for WGA, which would significantly improve genome coverage [39] Overall, this methodological approach opens new avenues in microbiology to interrogate virus–host pairs in the environment.

## Supplementary Material

SOM_Martinez-Garcia_clean_final_ycaf123

Supplementary_Data_1_final_ycaf123

## Data Availability

Data are available at Genbank SRA repository under the following Bioproject accession number PRJNA1246059. Assembled contigs from SPCs are available as Supplementary Data 1.

## Disclaimer

This report was prepared as an account of work sponsored by an agency of the United States Government. Neither the United States Government nor any agency thereof, nor any of their employees, makes any warranty, express or implied, or assumes any legal liability or responsibility for the accuracy, completeness, or usefulness of any information, apparatus, product, or process disclosed, or represents that its use would not infringe privately owned rights. Reference herein to any specific commercial product, process, or service by trade name, trademark, manufacturer, or otherwise does not necessarily constitute or imply its endorsement, recommendation, or favoring by the United States Government or any agency thereof. The views and opinions of authors expressed herein do not necessarily state or reflect those of the United States Government or any agency thereof.
