# Supplementary material for: Targeted genomic analysis of a predominant uncultured marine pelagiphage-host model via microfluidics and semipermeable capsule technology
Source: ISME Commun. 2025 Jul 17;5(1):ycaf123. doi: 10.1093/ismeco/ycaf123 (PMC12404659; doi:10.1093/ismeco/ycaf123)
Supplement: SOM_Martinez-Garcia_clean_final_ycaf123 [file som_martinez-garcia_clean_final_ycaf123.docx]

**Supplementary Material for**

**Targeted genomic analysis of a predominant uncultured marine pelagiphage-host model via microfluidics and semipermeable capsule technology**

Manuel Martínez-García^1,2*^, Monica Lluesma-Gomez^1,2*^, Laura Perez-Martin^2^, Esther Rubio-Portillo^2^, Ana Belen Martin-Cuadrado^2^, Francisco Nadal-Molero^2^, Aitana Escolano-Vico^2^, Fernando Santos Sanchez^2^, Victoria Orphan^3,^, and Josefa Antón^1,2*^

^1^Instituto Multidisciplinar para el Estudio del Medio Ramon Margalef, Parque Científico, Edificio Nuevos Institutos, University of Alicante, Ap- Correos 99 E-03690, San Vicente del Raspeig, Spain.

^2^Departament of Physiology, Genetics, and Microbiology, University of Alicante, Carretera de San Vicente s/n, 03080, San Vicente del Raspeig, Spain

^3^Division of Biology and Biological Engineering, California Institute of Technology, Pasadena, CA 91125, USA.

^4^Division of Geological and Planetary Sciences, California Institute of Technology, Pasadena, CA 91125, USA.

*Correspondence to: [m.martinez@ua.es](mailto:m.martinez@ua.es) and [anton@ua.es](mailto:anton@ua.es)

**This file contains methods, supplementary results, supplementary figures, table, data, and references**

**Methods**

**Samples encapsulated in SPCs**

**a) Pure culture of *Escherichia coli* K12 infected with bacteriophage T7.** *Escherichia coli* K12 was incubated at 37 ºC in Luria-Bertani (LB) broth (180 rpm shaking) and when culture reached exponential phase (DO 0.3), bacteriophage T7 was added to the culture to a MOI of 0.1. After 30 min of incubation with phage, infected *E. coli* culture was centrifuged at 6,000 g for 10 min, and the supernatant likely containing free phages was removed. Cell pellet was resuspended in sterile LB broth and processed for SPC encapsulation at a lambda ratio of 0.1 (10% of SPCs occupancy with single cells; see below for details).

**b) Culture of *E. coli* and *Vibrio coralliilyticus.*** *Escherichia coli* K12 was incubated at 37 ºC in Luria-Bertani (LB) broth and *Vibrio coralliilyticus* at 30ºC in LB broth with 3% NaCl, both cultures with 120 rpm shaking. The growth of the two bacteria was monitored by DAPI (4′,6-diamidino-2-phenylindole) counts and cells were diluted using a compatible buffer, sterile PBS 1x for *E.coli* and supplemented with 3% NaCl for *V. coralliilyticus*, to reach proper concentration in order to encapsulate the cells in semipermeable capsules (SPCs) at a lambda ratio of 0.1 (10% of SPCs occupancy with single cells).

**c) Seawater sample.** A total of 400 mL of surface seawater (≈0.5 m depth) was collected from the Cape Huertas, Mediterranean Sea (coordinates 38.35345133629738, −0.403225894080336; Alicante, Spain) at 03:00 pm on 11/02/2023. Sample was immediately transported to the laboratory in less than 20 min and processed as followed. Seawater was filtered through 0.45 μm using a polyether sulfone membrane filter (Millipore) for enriching SAR11 cells according to [1–3]. SAR11 cells and other ultrasmall marine species are highly enriched in the fraction of bacterioplankton cells that pass through 0.45-μm filters [1–3]. Then, sample was centrifuged at 30,000 g at 4ºC for 60 min to remove viruses present in the supernatant, and cell pellet was resuspended in 2 ml of sterile seawater. DAPI staining was performed [4] to estimate the cell abundance, and subsequent single bacterial cells were encapsulated in semipermeable capsules (SPCs) at a lambda ratio of 0.1 (10% of SPCs occupancy with single cells).

**d) Wastewater sample.** As a control**,** 50 ml of wastewater sample (input) from the wastewater treatment plant “Rincón de León” (Alicante city, Spain) was collected in October 10^th^ 2023. This wastewater sample was prefiltered by 20 μm, centrifuged at 6,000 g for 5 min at 4ºC, and resuspended in 1 ml of sterile PBS 1x buffer. Enumeration of cells was performed with DAPI, and then cells were encapsulated in SPCs at a lambda ratio of 1 (all SPC containing at least one single cell), to test efficiency of MDA-SPC in other natural complex samples different than seawater.

**Encapsulation of samples in SPCs**

Single cells from *E. coli* infected with phage T7 and seawater samples were encapsulated in SPCs at a lambda ratio of 0.1 (**~**90% of empty SPCs, 10.36% occupied with a single cell) using a first generation of SPC Chip Droplet Genomics CKP-G34 (Atrandi, Lithuania), which generated a total of one million of SPCs per lane (size 70 μm in diameter). Therefore, in those experiments, 900,000 SPCs were theoretically empty with no cells while a total of 100,000 SPCs contained one single cell with a frequency of SPCs containing more than one single cell of 0.56%. In the case of wastewater, cells were encapsulated as described above with the exception that we used a lambda ratio of 1.

For the encapsulation experiment of *E. coli* and *V. coralliilyticus,*  we used a second generation SPC Chip Droplet Genomics CKP-G34 (Atrandi, Lithuania), which generated a total of 200,000 SPCs per lane (size 80 μm in diameter) since microfluidic chip version 1 was discontinued by the company.

SPC microfluidic instrument version CHP-SPC1 was used in all experiments to generate the SPCs following manufacture’s protocol (Atrandi, Lithuania), except that SPC Generation Chip and Gaskets were previously UV-treated for 5 min in a UVP Ultraviolet CL-1000 Crosslinker as described in detail [5] in order to remove potential exogenous DNA, and that the sterile PBS 1x buffer used for sample dilution to achieve the desired lambda ratio was previously 16h UV-treated. Encapsulation was performed  in a laminar PCR hood (Alpina K1000) equipped with three ultraviolet lamps (18 W × 3) that sterilize the incoming flow air, which is dedicated exclusively for single-cell experiments [5, 6]. It is important to remark that before performing any work, we wiped down all clean hood surfaces, pipettes and equipment with 10% (wt/vol) bleach. We also UV-treated the clean hood for 60 min with equipment inside as described in [5]. In addition, all plastic material (e.g. Eppendorf’s and tubes used for SPC manipulation) used in our experiments were previously 16 h UV-treated as described [5], and operator was covered with individual protection equipment as in [5].

**Lysis, whole-genome amplification, and PCR in SPCs**

Once single cell encapsulation was completed, generated SPCs were fixed in cold 100% methanol and stored at -80ºC until use. Then, SPCs were thawed on iced and washed three times in 16h-UV treated sterile TE buffer 1x. In each washing step, SPCs were centrifuged at 1,000 g for 1 min, the supernatant was removed, and SPCs were resuspended again in 0.1 ml of the same sterile PBS 1x buffer. Then lysis was performed using a KOH shock following strictly protocols described for single-cell genomics [5]. In brief, SPCs were placed in 117 μl of 1h UV-treated sterile DLB buffer (Qiagen, Ref 1068797) containing KOH (pH14) at 4ºC for 10 min as described in [5], and then neutralization was performed with 1h UV-treated Qiagen Stop Solution (ref. 1032393) using 117 μl of volume. Then, one washing step as described above were performed before multiple-displacement amplification (MDA) carried out inside SPCs. We employ MDA conditions with EquiPhi 29 polymerase as described in [6] with no modifications. A total of 0.4 ml of MDA reaction buffer was used for each aliquot of 0.1 ml of SPCs. Real time MDA was monitored as in [6]. After MDA reaction, SPCs were washed three times with sterile PBS 1x buffer as described above. PCR was carried out with Phire Tissue Direct PCR (ThermoFisher, ref. 170S) according to manufacture´s manual. For each 0.1 ml volume of SPCs, a total of 0.2 ml of PCR reaction mix was used. PCR primers (500 nM final concentration) used in our experiments targeting different viruses and bacteria were fluorescently labelled at 5´end with fluorochrome Alexa488 and synthesized by IDT company (see the sequences at the end of this section). PCR primers used in our complementary experiment mixing *E. co*li K12 and *Vibrio coralliilyticus* SPCs at a ratio of 1:1, were designed targeting *Vibrio spp.* 16S rRNA gene. Only one fluorescently labelled primer was used in that case. All primers used in this study, except those targeting marine virus vSAG 37-F6, were designed with program Primer3 implemented in Geneious bioinformatic package [7]. Primers targeting capsid gene (ORF 9) of vSAG 37-F6 has been previously validated [8–11]. Primers targeting the human crassphage genome recently isolated from Barcelona (Spain) [12] was used as an external control in experiments.

PCR cycling conditions for primers targeting vSAG 37-F6 and T7 phage were as follows: 95ºC for 3 min, and 40 cycles of 95ºC for 15 sec, 62 ºC for 30 sec, and 60 ºC for 1 min extension. The rest of PCRs were carried out as follows: an initial activation step at 98C for 5 min, followed by 40 cycles of denaturation at 98ºC for 20 s, annealing at 62ºC for 5 s, and extension at 72ºC for 20 s, and a final extension at 72ºC for 1 min.

After PCR, SPCs were washed five times as described above, and then detection and sorting of positive fluorescently labelled SPCs were carried out with either Styx microfluidic sorter or Copas sorter instrument as described in the next section. It is important to note that between each molecular step in the overall workflow (encapsulation, first round of MDA, PCR, sorting, and second round of MDA), SPCs were thoroughly washed up to five times with sterile UV-treated buffer commonly used in SCGs workflow [5], to prevent undesired amplification of non-target DNA (e.g. carry over of leaked DNA fragments from the SPCs, DNA present outside SPCs, or from any other external contaminant sources, including the sheath fluid used during SPC sorting; see supplementary methods).

Primers used in this study:

-T7 phage forward primer AlexaFluo488 5´ ATGGCTGCTCGCTAAACAAG 3´

-T7 phage reverse primer AlexaFluo488 5´ CATGCTTTGTCTCCCTCAGC 3´

-Virus vSAG 37-F6 forward primer AlexaFluo488 5´ TTGCCAGTGTCGAAATCACC 3´

-Virus vSAG 37-F6 reverse primer AlexaFluo488 5´ TACACTCACGAGACCGTAGC 3´

-*Vibrio spp.* forward primer AlexaFluo488 5’GGATAACYATTGGAAACGATG 3’

-*Vibrio spp*. reverse primer 5’GAAATTCTACCCCCCTCTATAG 3’

-Virus crassphage forward primer AlexaFluo488 5´ TGTGTGCTTGACCCAACAAG 3´

-Virus crassphage reverse primer AlexaFluo488 5´ATCAGGGTCAGCCAAGTTCA 3´

**SPC sorting and downstream molecular analyses**

Sorting of positive PCR fluorescently labeled SPCs containing *E.coli* infected cells with phage T7 was carried out either in a Styx microfluidic sorter (Atrandi) or a Copas 500 (Unión Biometrica, USA) sorter instrument according to manufacture´s protocol. 488 nm laser was used for excitation and the Green channel was set to detect the emitted fluorescence in a range of 500-550 nm, targeting Alexa488 dye. The flow speed was adjusted to have an event frequency of 774 Hz. A total of 26,291 SPCs were screened and a total 500 positive SPCs were bulk sorted. In the case of sorting performed in a Copas biosorter FP100 instrument, we followed manufacture´s protocol and methods described in [13]. Sorted SPCs using Styx sorter were used for sequencing, while sorted SPCs using Copas instrument were employed for PCR screening in an electrophoresis gel (See fig. 1C). After sorting, SPCs were washed five times in 16h-UV treated PBS 1x sterile buffer. Then, SPCs were dissolved according to manufacture´s protocol kit ref. Droplet Genomics CKP-G34 (Atrandi, Lithuania) to release the bulk DNA, which was subjected to a second round of MDA as described above to generate enough DNA template for sequencing. Sequencing libraries were prepared with Illumina DNA Prep (ref. 20060059) and sequencing performed in a NextSeq Illumina sequencer.

Different negative and positive controls were performed in our experiments to corroborate that the positive observed fluorescence signal was observed only in the presence of the targeted microorganism (see Fig. S4). Additionally, a control experiment mixing cells of *E.coli* k12 and *V. coralliilyticus* (ratio 1:1) to monitor the sorting reliability was performed. For that experiment, 10 single PCR fluorescently labeled SPCs were sorted using Copas biosorter FP100 instrument. After DNA recovery and MDA amplification, each sample was subjected into PCR amplification using non-labelled *Vibrio sp.* primers to check the positive sorting ratio. PCR mixture included 2.5 µL of 10× PCR reaction buffer, 1 unit of Taq polymerase and 0.75 µL of 50 mM MgCl_2_ (Invitrogen, ref. 10342020), 0.75 µL of 10 mM dNTP mixture (Invitrogen ref. 10297-018), 1.25 µL of 10 µM (each) primer, 1 µL 1:10 diluted MDA-product amplification and sterile MilliQ water up to 25 µL. The PCR program was: 3 min at 94ºC followed by 35 cycles of 45 s at 94ºC, 1 min at 60ºC and 2 min at 72ºC, lastly a final extension of 10 min at 72ºC. The PCR products were analyzed by 1% agarose gel electrophoresis in 1x tris-acetate-EDTA (TAE) buffer (Fig. S1).

**Bioinformatic analysis of sequenced DNA from SPCs**

Illumina reads were trimmed using Trimmomatic program v0.36 [14] with default parameters. Taxonomic assignment of reads was carried out with Kaiju program v1.10.1 [15] using the Genbank nr as database for assignment. Genome mapping of reads against reference genomes (*E. coli, T7 phage, vSAG 37-F6)* was performed with Bowtie2 program v2.5.4 [16] by default parameters implemented in Geneious bioinformatic package [7]. Assembly was performed with SPAdes version 3.6.1 [17] by applying the following parameters: --sc, -k 33,55,77,99,127, --careful. ORFs were predicted with Prodigal [18] and Genmark [19], and gene annotation with DRAMv [20] implemented in Kbase platform [21]. Viral protein sharing network was performed as described in [6]. Alignment of capsid protein was carried out with Muscle aligner implemented in Geneious program. Binning of assembled contigs into metagenome-assembled genome (MAG) representing the targeted host was carried out with Maxbin2 [22] and MetaBAT2 [23], and carefully refined with DAS Tools [24] . Viral contigs were identified with Virsorter2 [25].

**Supplementary results and discussion**

**Validation of SPC technology with culture models**

To validate SPC microfluidic technology for virus-host interaction, we used a known cultured virus-host model. In a standard culture of *Escherichia coli* K12 infected with bacteriophage T7, single cells were encapsulated in SPCs (lambda ratio of 0.1; Fig. 1A), and after the described MDA and PCR procedure (Fig. 1B), we sorted 774 fluorescently labeled SPCs using either a microfluidic sorting Styx instrument [26](Fig. 1C) or a flow cytometry sorter (Fig. 1C) designed to target big particles and microorganisms [27] (Copas flow-sorting system; Union Biometrica). After sorting, SPCs were dissolved to release the bulk DNA, subjected to a second round of MDA, and Illumina sequenced (Table S1). Data indicate that the genome recovery was complete for the virus and nearly complete for the host (>99% breath coverage and nucleotide identity; Fig. 1D) with no detectable contamination from other DNA sources (see bar plot in Fig. 1D), such as human DNA in non-sterile microfluidic chips. In addition, the expected PCR product targeting the viral capsid gene from sorted positive PCR-SPCs containing putatively infected cells was obtained (Fig. 1C). Furthermore, in a complementary experiment, we assessed the purity and sensitivity of the Copas sorter instrument, later used for the screening of SPCs from a natural marine community. For that, we mixed *E. co*li and *Vibrio coralliilyticus* at a ratio of 1:1, encapsulated the cells (lambda ratio 0.1), performed MDA, and PCR screened with primers targeting *V. coralliilyticus*. Screening of ~10 individual sorted positive SPCs containing *Vibrio* cells resulted in positive PCR amplification in nearly all cases (8 out of 10 sorted SPCs; Fig. S1).

**SPC controls used for targeting cells with virus vSAG 37-F6**

A variety of six different controls were implemented to confirm that the observed fluorescence signal from positive SPCs were indeed only detected in the presence of the target virus (see Fig. S4). For instance, PCR screening of the marine sample was performed with specific primers for bacteriophage T7 and human crassphage. In addition, PCR screening of SPCs without cell encapsulation with different primer sets was carried out to rule out non-specific fluorescence signal. Finally, PCR screening of SPCs containing only vSAG 37-F6 genome as positive control was included as well. See results in Fig. S4.

**Future improvements**

In our study, the sorting and downstream analysis was not performed at the single-cell level limiting our ability to achieve fine-scale host range resolution and to study putative co-infections or the presence of multiple viruses within the same cell. This is particularly relevant given our intriguing observation of both vSAG 37-F6-like viruses and other viruses belonging to a mixed viral cluster, which did not contain the targeted sequence of vSAG 37-F6 primers. Co-location or co-sorting of non-specific viruses cannot be ruled out, albeit the co-occurrence of different viruses within the same cells has been previously reported [28]. Future improvements to this workflow could include the implementation of primary template-direct amplification for WGA, which would significantly improve genome coverage [29]and the use of a single round of WGA. So far, Illumina libraries directly generated from the bulk DNA released from a similar pool of sorted SPCs has been unsuccessful requiring an additional round of WGA. Furthermore, using smaller SPCs (approximately 30 μm) compatible with most state-of-the-art flow cytometer sorters with 384-well plates would enable high-throughput single-cell level screening.

**Supplementary Figures**


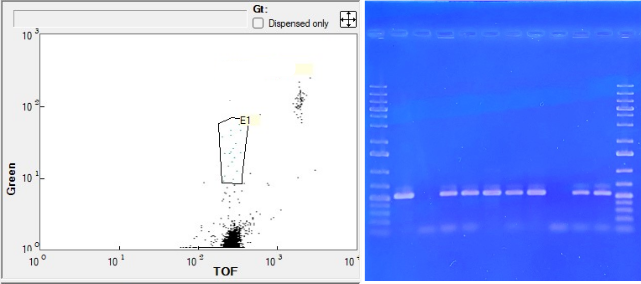


**Figure S1.** Experiment of mixing *E. coli* and *V. coralliilyticus*, SPC encapsulation, MDA, PCR and SPC sorting. Detection and sorting of positive PCR-SPCs with microfluidic Copas sorter and conventional agarose electrophoresis of amplicons from the PCR check of the sorted 10 single-SPC. The expected number of positive SPCs should be low given the percentage of occupancy.


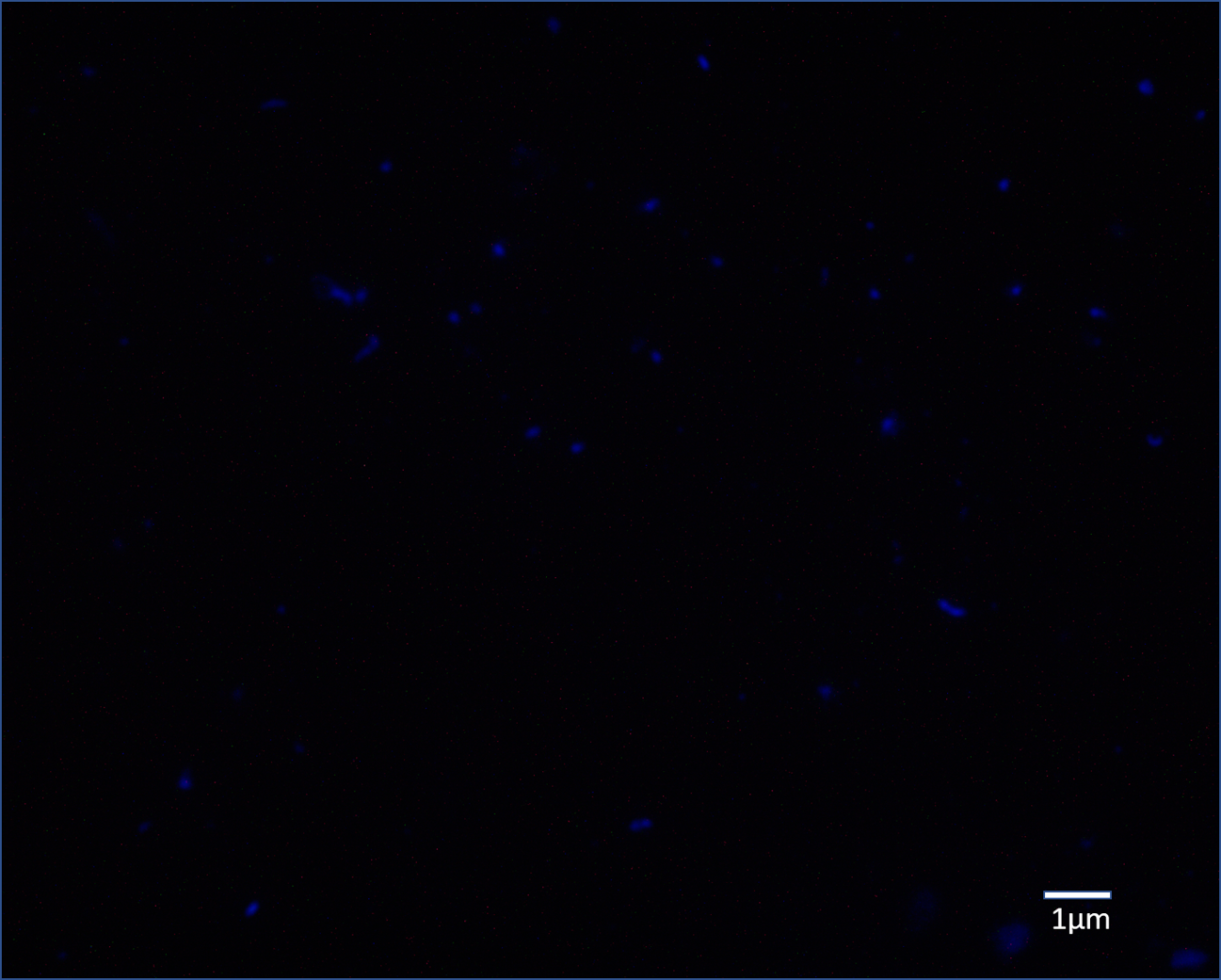


**Figure S2.** DAPI stain of cells (fraction ≤0.45 μm) before SPC encapsulation.

**
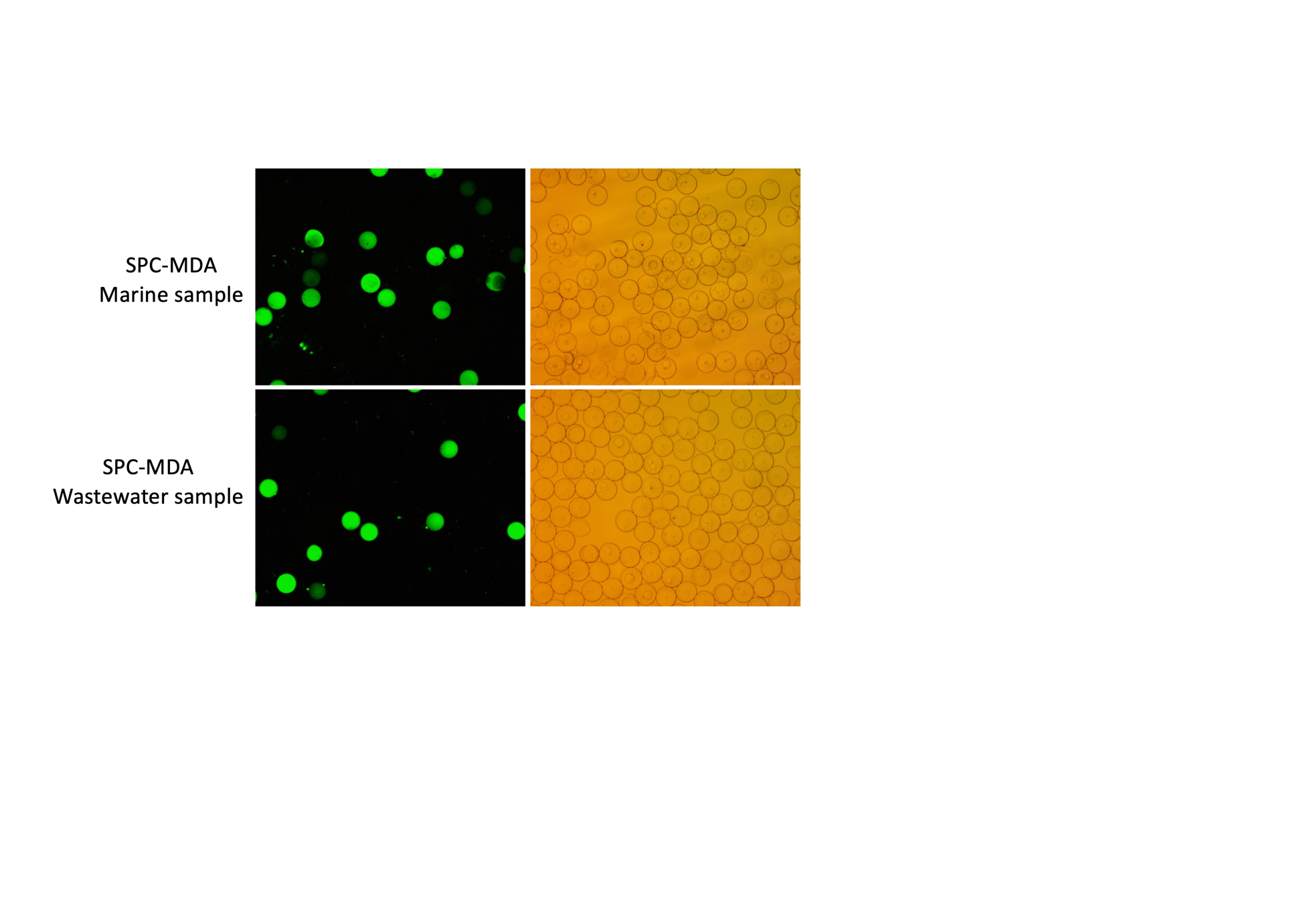
**

**Fig. S3.** Comparison of MDA rates between a marine sample collected in Cape Huertas processed as described in Methods and in main text (lambda encapsulation rate of 0.1; 10% of SPC occupancy) and a wastewater sample (input) collected from Rincón de León Wastewater Treatment Plant (Alicante city, Spain). This wastewater sample was prefiltered by 20 μm, and then encapsulated in SPC at a lambda ratio of 1 (all SPC containing at least one single cell), to test efficiency of MDA-SPC in other natural complex samples. As observed, the number of positive SPCs in the wastewater sample was much lower than expected (less than 8% of total SPCs yielded positive MDA; which is far from the expected value that should be around 100%), suggesting that MDA efficiency in seawater (around 10% of total SPC; close to expected theorical value) was close to be optimum, in contrast to wastewater SPC-MDA.


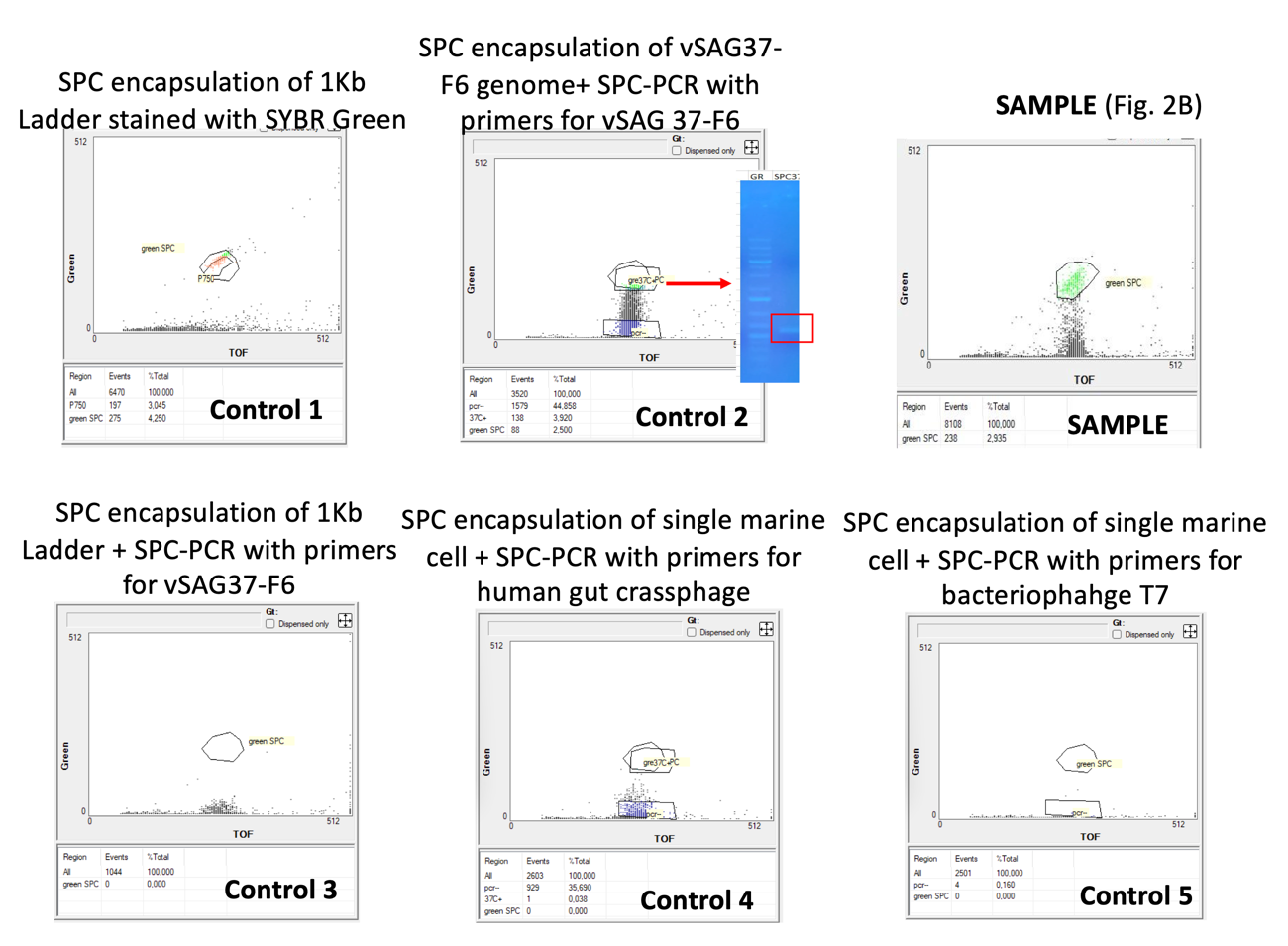


**Fig S4.** Controls used in SPC experiment to confirm that the observed green fluorescence signal was detected in the presence of the target microorganisms. Axis depict green fluorescence in relative units (Y axis), and Time of Flight that is a proxy of size particle (relative units; X axis). Different controls were included to corroborate that the observed green fluorescence signal was specific due to the presence of the target virus present in a single cell. In all cases the procedure was SPC encapsulation, MDA and SPC-PCR with different primers. In the case of control 2, expected SPC-PCR product of targeted capsid gene of virus vSAG 37-F6 was corroborated in an electrophoresis gel. For convenience, an additional control with encapsulation of mQ sterile water followed by MDA and PCR with the different primer sets used in our experiments is not shown since the resulting flow cytometry plots were very clean and similar to that of Control no. 5.

**Supplementary Table**

**Table S1**. Illumina sequencing data obtained from sorted positive SPC samples

| Sample | Sequencing data (Gbp) | No .of reads (size 150 bp) | Read quality  Q20 | Mean GC (%) |
| --- | --- | --- | --- | --- |
| ***E. coli* and bacteriophage T7** | 3.56 | 23704000 | 98.8% | 47.3 |
| **Marine bacteria and vSAG 37-F6** | 7.49 | 49608568 | 97.3 | 40 |

**Supplementary data**

**Supplementary Data 1** contains fasta sequences of contigs assembled from seawater sample analyzed by microfluidic SPC technology

**References**

1. Ghuneim LAJ, Jones DL, Golyshin PN, Golyshina O V. Nano-sized and filterable bacteria and archaea: Biodiversity and function. *Front Microbiol* 2018; **9**: 378457.

2. Ganesh S, Parris DJ, Delong EF, Stewart FJ. Metagenomic analysis of size-fractionated picoplankton in a marine oxygen minimum zone. *The ISME Journal advance online publication* 2013.

3. Giovannoni SJ. SAR11 Bacteria: The Most Abundant Plankton in the Oceans. *Ann Rev Mar Sci* 2017; **9**: 231–255.

4. Porter KG, Feig YS. The use of DAPI for identifying and counting aquatic microflora1. *Limnol Oceanogr* 1980; **25**: 943–948.

5. Rinke C, Lee J, Nath N, Goudeau D, Thompson B, Poulton N, et al. Obtaining genomes from uncultivated environmental microorganisms using FACS-based single-cell genomics. *Nat Protoc* 2014; **9**: 1038–1048.

6. Martinez-Hernandez F, Fornas O, Lluesma Gomez M, Bolduc B, de la Cruz Peña MJ, Martínez JM, et al. Single-virus genomics reveals hidden cosmopolitan and abundant viruses. *Nat Commun* 2017; **8**: 15892.

7. Kearse M, Moir R, Wilson A, Stones-Havas S, Cheung M, Sturrock S, et al. Geneious Basic: An integrated and extendable desktop software platform for the organization and analysis of sequence data. *Bioinformatics* 2012; **28**: 1647–1649.

8. Martinez-Hernandez F, Fornas Ò, Lluesma Gomez M, Garcia-Heredia I, Maestre-Carballa L, López-Pérez M, et al. Single-cell genomics uncover Pelagibacter as the putative host of the extremely abundant uncultured 37-F6 viral population in the ocean. *ISME Journal* 2019; **13**: 232–236.

9. McMullen A, Martinez‐Hernandez F, Martinez‐Garcia M. Absolute quantification of infecting viral particles by chip‐based digital polymerase chain reaction. *Environ Microbiol Rep* 2019; **11**: 1758-2229.12804.

10. Martinez-Hernandez F, Diop A, Garcia-Heredia I, Bobay LM, Martinez-Garcia M. Unexpected myriad of co-occurring viral strains and species in one of the most abundant and microdiverse viruses on Earth. *ISME J* 2022; **16**: 1025–1035.

11. Vila-Nistal M, Logares R, Gasol JM, Martinez-Garcia M. Time Series Data Provide Insights into the Evolution and Abundance of One of the Most Abundant Viruses in the Marine Virosphere: The Uncultured Pelagiphages vSAG 37-F6. *Viruses 2024, Vol 16, Page 1669* 2024; **16**: 1669.

12. Ramos-Barbero MD, Gómez-Gómez C, Sala-Comorera L, Rodríguez-Rubio L, Morales-Cortes S, Mendoza-Barberá E, et al. Characterization of crAss-like phage isolates highlights Crassvirales genetic heterogeneity and worldwide distribution. *Nature Communications 2023 14:1* 2023; **14**: 1–14.

13. Pulak R. Techniques for Analysis, Sorting, and Dispensing of C. elegans on the COPAS^TM^ Flow-Sorting System. *Methods Mol Biol* 2006; **351**: 275–286.

14. Bolger AM, Lohse M, Usadel B. Trimmomatic: a flexible trimmer for Illumina sequence data. *Bioinformatics* 2014; **30**: 2114–2120.

15. Menzel P, Ng KL, Krogh A. Fast and sensitive taxonomic classification for metagenomics with Kaiju. *Nat Commun* 2016; **7**: 11257.

16. Langdon WB. Performance of genetic programming optimised Bowtie2 on genome comparison and analytic testing (GCAT) benchmarks. *BioData Min* 2015; **8**: 1.

17. Bankevich A, Nurk S, Antipov D, Gurevich AA, Dvorkin M, Kulikov AS, et al. SPAdes: A New Genome Assembly Algorithm and Its Applications to Single-Cell Sequencing. *Journal of Computational Biology* 2012; **19**: 455–477.

18. Hyatt D, Chen GL, LoCascio PF, Land ML, Larimer FW, Hauser LJ. Prodigal: Prokaryotic gene recognition and translation initiation site identification. *BMC Bioinformatics* 2010; **11**.

19. Besemer J, Borodovsky M. GeneMark: web software for gene finding in prokaryotes, eukaryotes and viruses. *Nucleic Acids Res* 2005; **33**: W451.

20. Shaffer M, Borton MA, McGivern BB, Zayed AA, La Rosa SL 0003 3527 8101, Solden LM, et al. DRAM for distilling microbial metabolism to automate the curation of microbiome function. *Nucleic Acids Res* 2020; **48**: 8883–8900.

21. Arkin AP, Cottingham RW, Henry CS, Harris NL, Stevens RL, Maslov S, et al. KBase: The United States Department of Energy Systems Biology Knowledgebase. *Nature Biotechnology 2018 36:7* 2018; **36**: 566–569.

22. Wu Y-W, Simmons BA, Singer SW. MaxBin 2.0: an automated binning algorithm to recover genomes from multiple metagenomic datasets. *Bioinformatics* 2016; **32**: 605–607.

23. Kang DD, Li F, Kirton E, Thomas A, Egan R, An H, et al. MetaBAT 2: An adaptive binning algorithm for robust and efficient genome reconstruction from metagenome assemblies. *PeerJ* 2019; **2019**: e7359.

24. Sieber CMK, Probst AJ, Sharrar A, Thomas BC, Hess M, Tringe SG, et al. Recovery of genomes from metagenomes via a dereplication, aggregation and scoring strategy. *Nat Microbiol* 2018; **3**: 836–843.

25. Guo J, Bolduc B, Zayed AA, Varsani A, Dominguez-Huerta G, Delmont TO, et al. VirSorter2: a multi-classifier, expert-guided approach to detect diverse DNA and RNA viruses. *Microbiome* 2021; **9**: 1–13.

26. Breukers J, Ven K, Verbist W, Rutten I, Lammertyn J. From specialization to broad adoption: Key trends in droplet microfluidic innovations enhancing accessibility to non-experts. *Biomicrofluidics* 2025; **19**: 21302.

27. Pulak R. Techniques for analysis, sorting, and dispensing of C. elegans on the COPAS flow-sorting system. *Methods Mol Biol* 2006; **351**: 275–286.

28. Munson-Mcgee JH, Peng S, Dewerff S, Stepanauskas R, Whitaker RJ, Weitz JS, et al. A virus or more in (nearly) every cell: ubiquitous networks of virus–host interactions in extreme environments. *ISME J* 2018; **12**: 1706.

29. Gonzalez-Pena V, Natarajan S, Xia Y, Klein D, Carter R, Pang Y, et al. Accurate genomic variant detection in single cells with primary template-directed amplification. *Proc Natl Acad Sci U S A* 2021; **118**: e2024176118.
